# Structural and Biochemical Characterization of EFhd1/Swiprosin-2, an Actin-Binding Protein in Mitochondria

**DOI:** 10.3389/fcell.2020.628222

**Published:** 2021-01-18

**Authors:** Sang A. Mun, Jongseo Park, Kyoung Ryoung Park, Youngjin Lee, Jung Youn Kang, Taein Park, Minwoo Jin, Jihyeong Yang, Chang-Duk Jun, Soo Hyun Eom

**Affiliations:** ^1^School of Life Sciences, Gwangju Institute of Science and Technology, Gwangju, South Korea; ^2^Steitz Center for Structural Biology, Gwangju Institute of Science and Technology, Gwangju, South Korea; ^3^NuclixBio, Seoul, South Korea; ^4^Infection and Immunity Research Laboratory, Metabolic Regulation Research Center, Korea Research Institute of Bioscience and Biotechnology, Daejeon, South Korea; ^5^Department of Chemistry, Gwangju Institute of Science and Technology, Gwangju, South Korea

**Keywords:** EFhd1, swiprosin-2, crystal structure, β-actin, actin-binding protein, actin-bundling protein

## Abstract

Ca^2+^ regulates several cellular functions, including signaling events, energy production, and cell survival. These cellular processes are mediated by Ca^2+^-binding proteins, such as EF-hand superfamily proteins. Among the EF-hand superfamily proteins, allograft inflammatory factor-1 (AIF-1) and swiprosin-1/EF-hand domain-containing protein 2 (EFhd2) are cytosolic actin-binding proteins. AIF-1 modulates the cytoskeleton and increases the migration of immune cells. EFhd2 is also a cytoskeletal protein implicated in immune cell activation and brain cell functions. EFhd1, a mitochondrial fraternal twin of EFhd2, mediates neuronal and pro-/pre-B cell differentiation and mitoflash activation. Although EFhd1 is important for maintaining mitochondrial morphology and energy synthesis, its mechanism of action remains unclear. Here, we report the crystal structure of the EFhd1 core domain comprising a C-terminus of a proline-rich region, two EF-hand domains, and a ligand mimic helix. Structural comparisons of EFhd1, EFhd2, and AIF-1 revealed similarities in their overall structures. In the structure of the EFhd1 core domain, two Zn^2+^ ions were observed at the interface of the crystal contact, suggesting the possibility of Zn^2+^-mediated multimerization. In addition, we found that EFhd1 has Ca^2+^-independent β-actin-binding and Ca^2+^-dependent β-actin-bundling activities. These findings suggest that EFhd1, an actin-binding and -bundling protein in the mitochondria, may contribute to the Ca^2+^-dependent regulation of mitochondrial morphology and energy synthesis.

## Introduction

Regulation of the cytoskeleton is essential for cell dynamics, such as the maintenance of cell shape or motility (Egelman, 2004; Wu et al., 2016). Its malfunction promotes muscle weakness, cerebral arteriopathy, cardiomyopathy, and brain abnormalities (Parker et al., 2020). As the major cytoskeletal protein is actin, its regulation is responsible for several cellular functions, including maintenance of cellular morphology and formation of lamellipodia or filopodia (Lee and Dominguez, 2010). In the cytosol, actin monomers form actin filaments, and the actin filament networks are modulated by several actin-binding proteins (ABPs), including profilin and cofilin, which regulate the polymerization of actin and actinin, fascin, allograft inflammatory factor-1 (AIF-1), and EF-hand domain-containing protein 2 (EFhd2), which facilitate actin-bundling or cross-linking (Dubernard et al., 1997; Autieri et al., 2003; Aratyn et al., 2007; Lee and Dominguez, 2010; Kwon et al., 2013; Ali et al., 2016). In the mitochondria, the maintenance of morphology and function requires the mitochondrial actin, β-actin (Xie et al., 2018). β-actin knockout (KO) in mitochondria induces a severe loss of mitochondrial membrane potential, resulting in impaired mitochondrial DNA transcription and large aggregates of nucleoids (Xie et al., 2018). The EF-hand domain-containing protein 1 (EFhd1), a homologous protein of EFhd2, is localized in the mitochondria (Tominaga et al., 2006; Dutting et al., 2011). Since the gene encoding EFhd1 is not present in the mitochondrial DNA, following its translation in cytoplasm, EFhd1 translocates from the cytoplasm to the mitochondria (Anderson et al., 1981).

EFhd2, AIF-1, and EFhd1 have Ca^2+^-binding EF-hand motifs, which belong to the EF-hand superfamily, but they have distinct subcellular locations (Dutting et al., 2011). For cytosolic EF-hand superfamily, EFhd2 was first identified in lymphocytes, and it regulates cell spreading and the cell migration of immune and epithelial cells by F-actin rearrangement (Vuadens et al., 2004; Aratyn et al., 2007; Ramesh et al., 2009; Kwon et al., 2013). The crystal structure of Ca^2+^-bound state and EF-hand mutants of EFhd2 have been reported previously (Park et al., 2016). The overall structures of EFhd2 are compact and rigid, comparable to those of Ca^2+^-calmodulin-peptide complexes; however, EF-hand motifs are flexible in the mutant structures. Since the rigidity of EF-hand motifs in EFhd2 is essential for the F-actin-bundling activity of EFhd2, the mutants cannot bundle F-actin *in vitro* (Park et al., 2016; Durvanger and Harmat, 2019). AIF-1 is another cytosolic ABP that induces F-actin-bundling to control membrane ruffling in immune cells (Sasaki et al., 2001; Kanazawa et al., 2002; Autieri et al., 2003). The crystal structure of AIF-1 has a similar overall topology to that of EFhd2 (Yamada et al., 2006; Park et al., 2016). Unlike EFhd2 and AIF-1, EFhd1 is localized in the mitochondria and may regulate mitochondrial energy metabolism (Tominaga et al., 2006). EFhd1 modulates the apoptosis and differentiation of neuronal and muscle cells (Tominaga et al., 2006; Dutting et al., 2011). In addition, EFhd1 induces not only mitoflashes but also metabolic changes during the development of pro-/pre-B cells (Hou et al., 2016; Stein et al., 2017). A recent report suggested that EFhd1 affects mitochondrial morphology and energy production in the dorsal root ganglion neurons (Ulisse et al., 2020). However, the mechanism underlying the regulation of how EFhd1 regulates several cellular functions is currently unclear.

Here, we report the crystal structure of the core domain of mouse EFhd1 (_CD_EFhd1, residues 79–180) in a Ca^2+^-bound state, comprising a proline-rich (PR) region, two EF-hand motifs, a ligand mimic helix (LM-helix), and a C-terminal linker. The overall structure of _CD_EFhd1 was similar to that of _CD_EFhd2 and AIF-1. Intriguingly, we found two Zn^2+^ ions in the crystal packing interface, suggesting the plausible Zn^2+^-mediated multimerization. In addition, we identified Ca^2+^-independent α- and β-actin-binding and Ca^2+^-dependent β-actin-bundling activities of EFhd1, indicating that EFhd1 might be involved in the Ca^2+^-dependent regulation of mitochondrial morphology via interactions with β-actin.

## Materials and Methods

### Cloning, Expression, and Purification of EFhd1 ΔNTD (Residues 69–240)

The mouse EFhd1 ΔNTD was amplified from full-length EFhd1 using polymerase chain reaction (PCR). The amplified DNA was cloned into a modified pET-28a vector (Novagen) that carried an N-terminal 6 × His (His_6_)-tobacco etch virus (TEV) protease cleavage site (Glu-Asn-Leu-Tyr-Phe-Gln/Gly). The recombinant plasmid was transformed into *Escherichia coli* strain BL21 (DE3) cells for protein expression. The cells were cultured at 37°C in Luria-Bertani (LB) broth containing 50 μg/mL kanamycin until the absorbance at 600 nm reached 0.7. Recombinant protein expression was induced with isopropyl β-D-1-thiogalactopyranoside (IPTG) (final concentration of 0.5 mM), and the cells were cultivated for an additional 4 h at 37°C. Cells were harvested by centrifugation at 4,000 × *g* for 15 min at 4°C. The harvested cell pellet was suspended in a lysis buffer [50 mM HEPES-NaOH pH 7.5, 300 mM NaCl, 20 mM imidazole, 0.4 mM phenylmethylsulfonyl fluoride (PMSF), and 14.3 mM β-mercaptoethanol]. The resuspended cells were disrupted via sonication and centrifuged at 14,000 × *g* for 50 min at 4°C to discard cell debris. The soluble supernatant was loaded onto a gravity-flow column (Bio-Rad, Hercules, CA, USA) packed with Ni-IDA agarose resin (Elpis), pre-equilibrated, and subsequently washed with the lysis buffer to remove any non-specific proteins. The desired protein was eluted with lysis buffer supplemented with 400 mM imidazole. After concentrating the eluate, the protein solution was incubated with TEV protease overnight at 4°C to cleave the N-terminal His_6_-TEV tag. To exchange the buffer for crystallization, the final purified protein was passed through a HiLoad 16/60 Superdex 75 gel-filtration column (Pharmacia Biotech) pre-equilibrated with the final buffer (20 mM HEPES-NaOH pH 7.5, 150 mM NaCl, 0.4 mM PMSF, and 14.3 mM β-mercaptoethanol).

The purified protein was concentrated using a 10 K centrifugal filter (Millipore) and stored at −80°C. During purification, the presence of EFhd1 protein was confirmed via sodium dodecyl sulfate-polyacrylamide gel electrophoresis (SDS-PAGE), and protein degradation was observed following incubation with TEV protease.

### Cloning, Expression, and Purification of Full-Length EFhd1 and EFhd2

To investigate the actin-binding function, we purified full-length EFhd1 and EFhd2. Full-length EFhd1 was amplified using PCR and cloned into the modified pET-28a vector (Novagen) carrying an N-terminal His_6_-TEV tag. The overall expression and affinity chromatography procedure of full-length EFhd1 was similar to that of EFhd1 ΔNTD, except for the step involving the incubation of TEV protease. The process for the removal of the N-terminal His_6_-TEV tag was omitted due to protein degradation. After affinity chromatography, the eluted protein was concentrated. The final purified protein was passed through a HiLoad 16/60 Superdex 75 gel-filtration column pre-equilibrated with the final buffer (20 mM HEPES-NaOH pH 7.5, 150 mM NaCl, 0.8 mM PMSF, and 5 mM DTT). The purified protein was concentrated using 10 K centrifugal filters (Millipore) and stored at −80°C.

Full-length EFhd2 was amplified using PCR and cloned into the modified pET-28a vector carrying an N-terminal His_6_ tag. The recombinant plasmid was transformed into *E. coli* strain BL21 (DE3) cells to express the protein. The cells were grown at 37°C in LB broth containing 50 μg/mL kanamycin until the absorbance at 600 nm reached 0.7. The recombinant protein was induced with 0.5 mM IPTG and the cells were cultured for an additional 5 h at 37°C. Cells were harvested via centrifugation at 4,000 × *g* for 15 min at 4°C, and the harvested cell pellet was suspended in a lysis buffer (50 mM HEPES-NaOH pH 7.5, 300 mM NaCl, 5 mM imidazole, 0.4 mM PMSF, and 14.3 mM β-mercaptoethanol). The resuspended cells were disrupted via sonication and centrifuged at 14,000 × *g* for 50 min at 4°C to remove cell debris. The soluble supernatant was loaded onto a gravity-flow column packed with Ni-IDA agarose resin previously equilibrated and subsequently washed with the lysis buffer to remove any non-specific proteins. The protein was eluted with lysis buffer supplemented with 400 mM imidazole. After concentrating the eluate, the final purified protein was passed through a HiLoad 16/60 Superdex 75 gel-filtration column pre-equilibrated with the final buffer (20 mM HEPES-NaOH pH 7.5, 150 mM NaCl, 0.8 mM PMSF, and 5 mM DTT). The purified protein was concentrated using 10 K centrifugal filters (Millipore) and stored at −80°C.

### Crystallization of EFhd1 Core Domain (Residues 79–180)

Initially, we attempted to crystallize Ca^2+^-bound EFhd1 ΔNTD (residues 69–240). EFhd1 ΔNTD was incubated for at least 20 min on ice after the addition of 1 mM CaCl_2_ and then screened using the sitting-drop vapor-diffusion method in a 96-well INTELLI-PLATE (Art Robbins Ins.). We found that EFhd1 ΔNTD was degraded and the core domain (_CD_EFhd1, residues 79–180) was crystallized. _CD_EFhd1 formed rod-shaped crystals after 1 week in a reservoir solution containing 80 mM HEPES-NaOH (pH 7.0), 2 mM ZnSO_4_, and 25% (*v*/*v*) Jeffamine ED-2003 (Molecular Dimensions). Additional refinements of crystallization conditions were performed using the sitting-drop vapor-diffusion method, and drops were prepared by mixing 1 μL of protein and 1 μL of reservoir solution. Crystals were obtained using a reservoir solution containing 0.1 M HEPES-NaOH (pH 7.5), 5 mM ZnSO_4_, and 25% (w/*v*) Jeffamine ED-2001 (Hampton Research). For data collection, _CD_EFhd1 crystals were cryoprotected by transferring into a mother liquor containing additional 30% (*v*/*v*) glycerol and flash freezing in liquid nitrogen.

### X-ray Data Collection, Structure Determination, and Refinement

X-ray diffraction data of _CD_EFhd1 were collected at 100 K using synchrotron X-ray sources on beamline 5C at the Pohang Accelerator Laboratory (PAL, South Korea). We collected the best resolution diffraction data for _CD_EFhd1 at a 2.07 Å resolution. The _CD_EFhd1 crystal belongs to the space group *P*2_1_2_1_2_1_ with cell dimensions of *a* = 31.8, *b* = 47.6, and *c* = 87.2 Å. The diffraction data were indexed, processed, and scaled using the *HKL2000* suite (Otwinowski and Minor, 1997). Template for molecular replacement (MR) of the EFhd1 core domain was generated by the SWISS-MODEL homology-modeling server using the human EFhd2 core domain (PDB ID: 5I2L) as the template (Waterhouse et al., 2018). Using this homology-model, the initial model of EFhd1 was determined via MR using *Phaser* in CCP4 (McCoy et al., 2007; Winn et al., 2011). Using the initial model, additional model building was performed using the COOT program (Emsley and Cowtan, 2004). Iterative refinement was performed with *phenix.refine* (Afonine et al., 2012; Liebschner et al., 2019). The details of the data collection and refinement statistics are provided in Table 1.

**Table 1:** Data collection and refinement statistics.

**Data collection**	**_**CD**_EFhd1 (PDB ID: 7CLT)**
Space group	*P*2_1_2_1_2_1_
X-ray source^a^ and detector	PAL-5C Pilatus 6M
Wavelength (Å)	0.9794
Unit cell: *a, b, c* (Å)	31.8, 47.6, 87.2
*α, β, γ* (°)	90.0, 90.0, 90.0
Resolution range (Å)^b^	50–2.07 (2.11–2.07)
$R_{\text{merge}}^{\text{c}}$	4.6 (59.0)
${\text{CC}}_{1/2}^{\text{d}}$	0.998 (0.873)
<*I*/σ(*I*)>	11.1 (2.5)
Completeness (%)	99.2 (97.1)
Redundancy	4.9 (4.3)
**REFINEMENT**	
Resolution range (Å)	43.6–2.07
No. reflections	7896
$R_{\text{work}}^{\text{e}}$ (%)/*R*_free_ (%)	20.9/22.9
**NO. ATOMS (RESIDUES)**	
Protein	827 (102)
Glycerol	6 (1)
Ca^2+^	2
Zn^2+^	2
Water	23
**B-Factors (Å**^**2**^**)**	
Protein	31.8
Glycerol	22.4
Ca^2+^	20.8
Zn^2+^	24.0
Water	27.2
**MODEL STATISTICS**	
RMSD bond length (Å)	0.007
RMSD bond angles (°)	0.87
Ramachandran plot (%) favored/allowed/disallowed	98.0/2.0/0.0

a * Beamline 5C at Pohang Acceleratory Laboratory (PAL) in the Republic of Korea*.

b * Values in parentheses are for the highest resolution shell*.

c * R_merge_ = ∑_h_ = _i_ |I(h)_i_- < I(h) > |/ [∑_h_ /_i_I(h)_i_], where I(h) is the intensity of reflection of h, ∑_h_ is the sum over all reflections and -_i_ is the sum over i measurements of reflection h*.

d * CC_1/2_ was calculated from HKL2000*.

e * R_work_ = Σ_hkl_ ||F_o_|–|F_c_||/(Σ_hkl_|F_o_|); 5% of the reflections were excluded for the R_free_ calculation*.

### Structural Analysis

All structural figures were generated using *PyMOL* version 1.5.0.4 (Schrödinger LLC). *PDBePISA* was used to analyze the interface, and the *PRODIGY* web server was used to predict the binding energies of symmetry-mate molecules (Krissinel and Henrick, 2007; Xue et al., 2016). Multiple sequence alignment was performed using *ESPript* 3.0 (Robert and Gouet, 2014). The *F*_obs_-*F*_calc_ map was calculated using *phenix.maps* and converted to the ccp4 format using a *phenix.mtz2map* (Liebschner et al., 2019).

### Zn^2+^-Dependent Precipitation Assay

To measure the precipitation of EFhd1 and EFhd2 in various Zn^2+^ concentrations, we performed an *in vitro* precipitation assay. First, 6 μM of His_6_-TEV tagged full-length EFhd1 and His_6_ tagged full-length EFhd2 were incubated with 20 μM to 10 mM ZnCl_2_ in reaction buffer (100 mM KCl, 0.2 mM Tris-HCl, pH 8.0) at 24°C for 30 min. The precipitated proteins were pelleted via centrifugation at 15,000 × *g* for 10 min at 24°C. Equal volumes of pellet or supernatant solutions were resolved via SDS-PAGE, and the protein bands were visualized via Coomassie Brilliant Blue staining.

To measure the $K_{1/2}^{agg}$value (concentration of half maximal protein aggregation for Zn^2+^), we used a spectrophotometric method. First, 6 μM of His_6_-TEV tagged full-length EFhd1 and His_6_ tagged full-length EFhd2 were incubated with various concentrations of ZnCl_2_ (0–1 mM ZnCl_2_ with EFhd1, 0–20 mM ZnCl_2_ with EFhd2) in reaction buffer (100 mM KCl, 0.2 mM Tris-HCl, pH 8.0) at 24°C for 30 min. The turbidity of the reacted proteins was monitored by measuring the absorbance at 470 nm using a spectrophotometer (Ultrospec 2000; Pharmacia Biotech). Graphs of absorbance at 470 nm were fitted using *OriginPro 9.1* software (OriginLab Corporation, Northampton, MA, USA).

### *In vitro* Actin-Binding Assay

Actin co-sedimentation assays were performed as previously reported (Kwon et al., 2013). In brief, non-muscle actin (85% β-actin and 15% γ-actin), derived from human platelets, and muscle actin (α-actin), derived from rabbit skeletal muscle (Cytoskeleton Inc.), were mixed in G-buffer (0.2 mM CaCl_2_, 5 mM Tris-HCl, pH 8.0) to produce actin stock and polymerized in an actin polymerization buffer (100 mM KCl, 2 mM MgCl_2_, 0.5 mM ATP, 0.2 mM Tris-HCl, pH 8.0) at 24°C for 1 h. Solutions containing polymerized actin (8 μM) were incubated with bovine serum albumin (BSA, 4 μM), EFhd1 (12 μM), or EFhd2 (12 μM) for 30 min at 24°C in the presence of 1 mM ethylene glycol tetraacetic acid (EGTA) or 1 mM CaCl_2_. Actin filaments with each protein were pelleted via centrifugation at 100,000 × *g* for 2 h at 24°C (for the actin-binding assay). BSA and EFhd2 were used as negative and positive controls, respectively. Equal amounts of pellet and supernatant were resolved via SDS-PAGE, and the protein bands were visualized by Coomassie Blue staining. The percentage of each protein in the pellet was quantified via densitometry using *ImageJ 1.44p*, and the percentage of pellet histogram was plotted using *OriginPro 9.1* software (OriginLab Corporation, Northampton, MA, USA) (Schneider et al., 2012).

### Negative Staining Electron Microscopy Imaging

Non-muscle actin (Cytoskeleton Inc.) was polymerized in F-actin buffer containing 100 mM KCl, 2 mM MgCl_2_, 0.5 mM ATP, and 0.2 mM Tris-HCl at pH 8.0. Mixtures (50 μL) of F-actin (4 μM) and full-length EFhd1 (6 μM) in the presence of 1 mM EGTA or 0.5 mM CaCl_2_ were allowed to react for 1 h. For grid preparation, 2 μL of reaction mixture was loaded onto the Formvar and metal-coated grids and blotted with filter paper to remove excess samples. The sample-loaded grid was stained using a solution of 1% (*w*/*v*) uranyl acetate. The grids were immersed in the stain solution for 20 min, blotted with filter paper to remove excess stain, and air-dried. The samples were analyzed using an FEI Tecnai G2 transmission electron microscope operated at 120 kV.

## Results

### Overall Structure of the EFhd1 Core Domain in the Ca^2+^-Bound State

**Figure 1:**
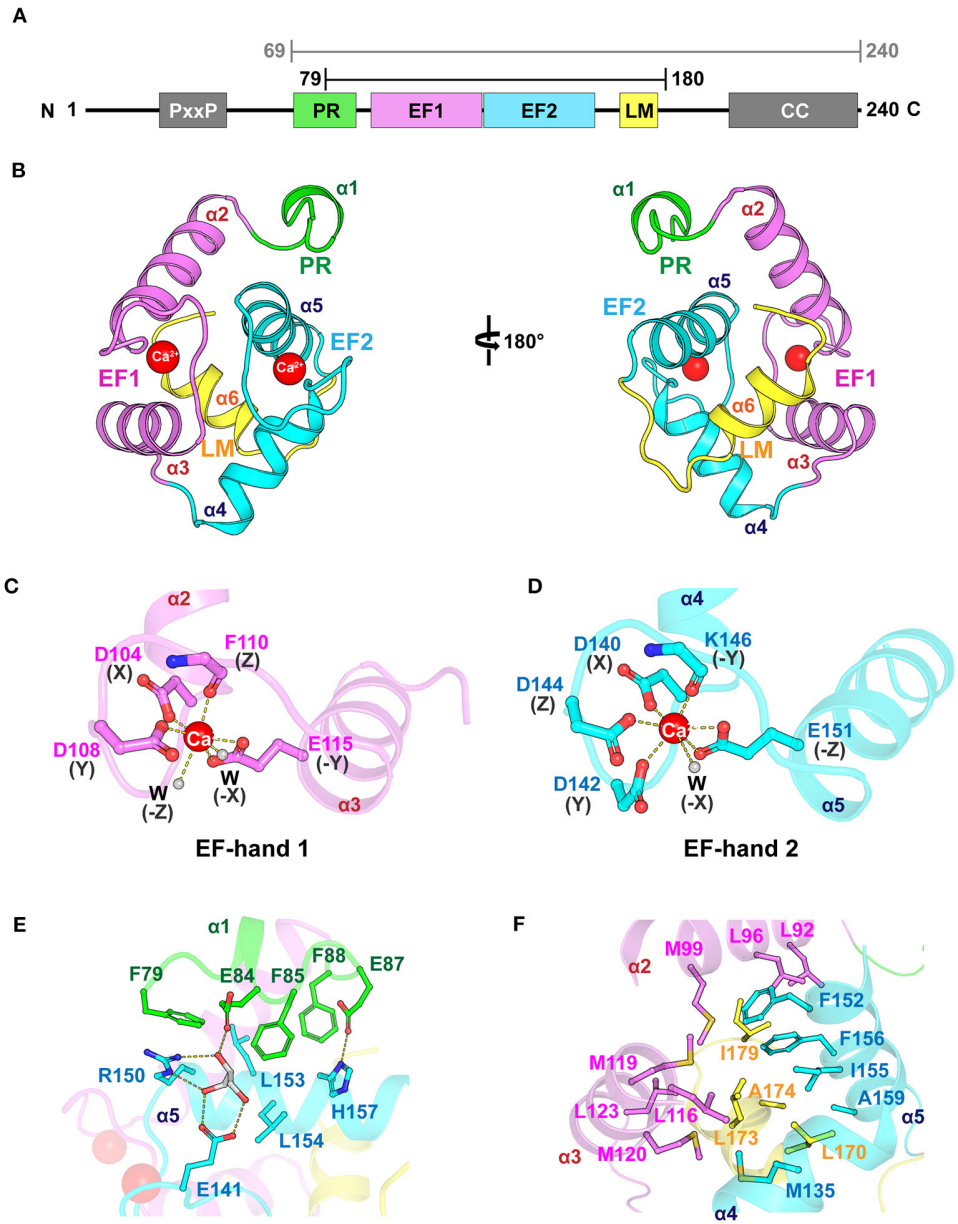
Overall structure of _CD_EFhd1. **(A)** Schematic diagram of mouse EFhd1 consisting of a PR (proline-rich) region, EF1 (EF-hand 1), EF2 (EF-hand 2), LM (ligand mimic)-helix, and CC (coiled coil). The upper bars indicate purified regions of EFhd1 (residues 69–240) and crystallized regions of EFhd1 (residues 79–180), respectively. **(B)** The overall structure of the core domain of EFhd1 (_CD_EFhd1). The PR region is colored green. EF1 and EF2 are colored violet and cyan, respectively. The LM-helix was colored yellow. **(C,D)** A cartoon representation of EF1 **(C)** and EF2 **(D)** with Ca^2+^ represented by a red sphere. The residues participating in Ca^2+^ coordination are represented in the stick form. Ca^2+^ coordination is marked by dashed lines. **(E)** Detailed view of the interaction between PR region and EF2. The interacting residues are represented in stick form, and the hydrogen bonds are marked by dashed lines. **(F)** Detailed view of the interaction between the LM-helix and two EF-hand motifs. The interacting residues are represented in stick form.

We determined the crystal structure of the core domain of mouse EFhd1 (_CD_EFhd1, residues 79–180) at a resolution of 2.07 Å and refined to *R*_work_ = 20.9 (%) and *R*_free_ = 22.9 (%) (Table 1). We initially attempted to crystallize the EFhd1 ΔNTD (residues 69–240) construct, but only the core domain was crystallized due to proteolytic degradation (Figure 1A). The _CD_EFhd1 structure comprised two EF-hand motifs (residues 91–162), an LM-helix (residues 169–176), a C-terminus of the PR region (residues 79–89) at the N-terminus, and a C-terminal linker (residues 177–180) (Figure 1B). Within the structure, Ca^2+^ ions were coordinated in each of the two EF-hand motifs of _CD_EFhd1 (Figures 1C,D). Consensus residues for Ca^2+^ coordination in the EF-hand consist of 12 amino acids with patterns of 1(X), 3(Y), 5(Z), 7(-Y), 9(-X), and 12(-Z) comprised of the five monodentate ligands and one bidentate ligand for -Z (Lewit-Bentley and Rety, 2000). Consequently, the geometry for Ca^2+^ coordination of the EF-hand is generally pentagonal bipyramid with a coordination number of seven comprising six oxygen atoms from the side chains and one main-chain carbonyl oxygen of -Y (Lewit-Bentley and Rety, 2000; Grabarek, 2006). In the case of EF-hand 1 of _CD_EFhd1, two water molecules participated in the Ca^2+^ coordination instead of the residues in position Y (G106) and -X (D112) (Supplementary Figure 1). Notably, this alternative pattern of Ca^2+^ coordination formed a distorted pentagonal bipyramid geometry. Unlike EF-hand 1, in the case of EF-hand 2 of _CD_EFhd1, one water molecule participated in Ca^2+^ coordination instead of the residues in position -X (S148). The Ca^2+^ coordination geometry of EF-hand 2 was maintained in the general pentagonal bipyramid. Collectively, EF-hand 1 and EF-hand 2 of _CD_EFhd1 had a distorted or general geometry for Ca^2+^ coordination, respectively.

The structure of the motifs in _CD_EFhd1 was stabilized by hydrophobic intramolecular interactions. In the PR region (helix α1), three Phe residues (F79, F85, and F88) formed hydrophobic interactions with L153 and L154 of helix α5 of EF-hand 2 (Figure 1E). The interaction of the PR region and helix α5 was further stabilized through the hydrogen bond network comprising E84 (PR region), E87 (PR region), E141 (interloop of EF-hand 2), R150 (helix α5), H157 (helix α5), and a glycerol molecule (gray). The LM-helix was stabilized by the intramolecular hydrophobic interaction network comprising the LM-helix (L170, L173, A174), helix α2 (M99), helix α3 (L116, M119, M120, L123), helix α4 (M135), and helix α5 (F152, I155, F156, A159) (Figure 1F). In the case of the C-terminal linker, I179 formed hydrophobic interactions with L92, L96 of helix α2, and F156 of helix α5. Collectively, _CD_EFhd1 formed a compact and rigid domain structure through these intramolecular interactions.

### Structural Comparison Between the Core Domain of EFhd1, EFhd2, and AIF-1

**Figure 2:**
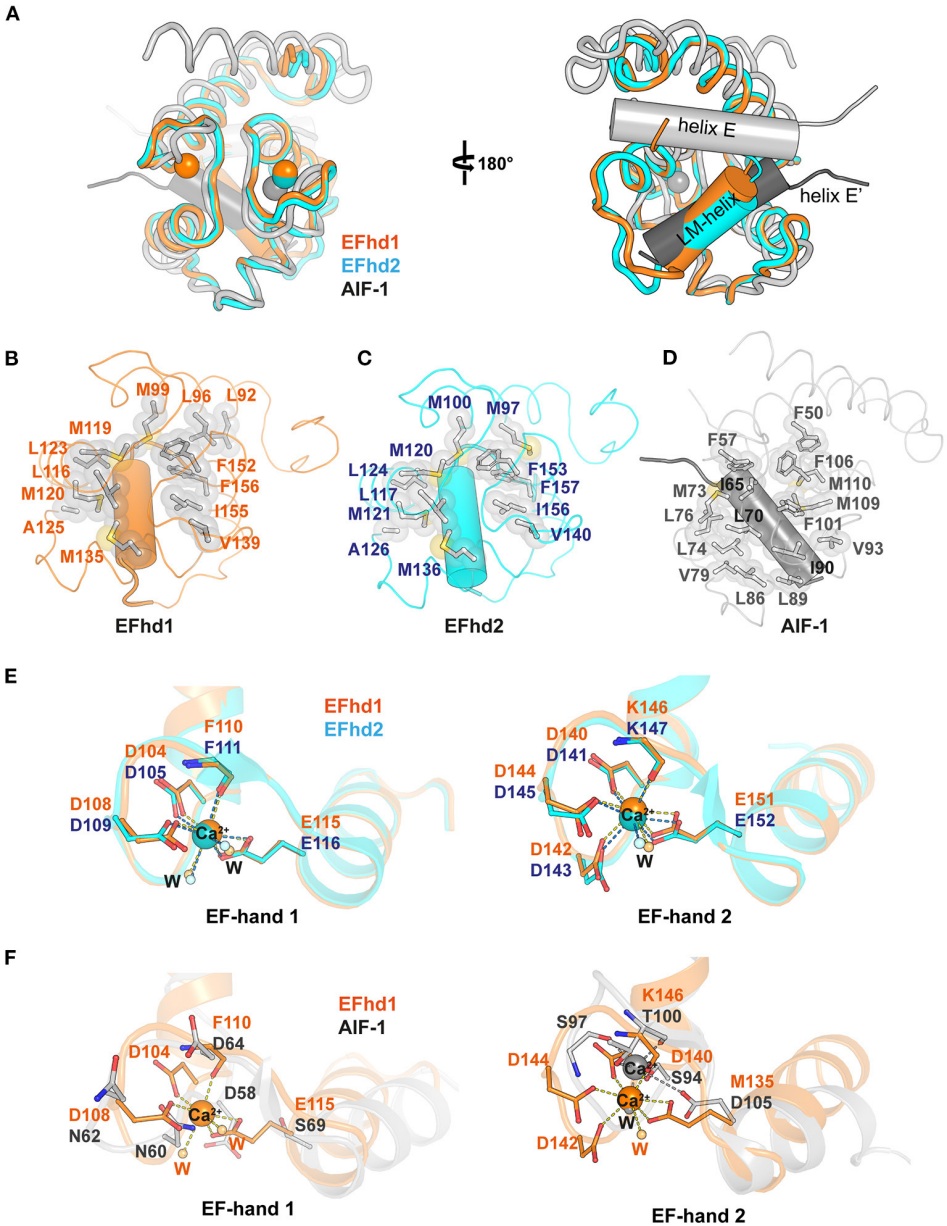
Structural comparison between Ca^2+^-bound _CD_EFhd1, _CD_EFhd2, and AIF-1. **(A)** Ribbon diagrams of superimposed _CD_EFhd1 (PDB ID: 7CLT), _CD_EFhd2 (PDB ID: 5I2L), and AIF-1 (PDB ID: 1WY9). The ribbon diagrams are represented in different colors: orange (_CD_EFhd1), cyan (_CD_EFhd2), and gray (AIF-1). The LM-helices of EFhd1 (orange) and EFhd2 (cyan) are presented in a cylindrical diagram. Helix E (gray) and E′ (dark gray) of AIF-1 are presented in the cylindrical diagram. Helix E′ is derived from the symmetry mate molecule of AIF-1. **(B–D)** Detailed view of the hydrophobic networks of the EFhd1 **(B)**, EFhd2 **(C)**, and AIF-1 **(D)**. The hydrophobic residues are represented in the stick and surface form. The LM-helices of EFhd1 and EFhd2 are colored in orange and cyan, respectively. The helix E′ in AIF-1 is colored in dark gray. The LM-helices and helix E′ are represented in the cylindrical diagram. **(E,F)** Detailed view of superimposed EF-hand 1 or EF-hand 2 on EFhd1 (orange) and EFhd2 (cyan) **(E)**, or EFhd1 (orange) and AIF-1 (gray) **(F)**. The residues for Ca^2+^ coordination are represented in the stick form. Ca^2+^ is marked by orange (EFhd1), cyan (EFhd2), or gray (AIF-1) spheres. Ca^2+^ coordination is marked by dashed lines.

The genes encoding EFhd1, EFhd2, and AIF-1 evolved from the common ancestral species of *Bilateria* (Dutting et al., 2011). The sequences of EFhd1 and EFhd2 are highly conserved, with a sequence identity of 65%, but the sequence of AIF-1 is conserved with that of EFhd1 only in the EF-hand motifs with an overall sequence identity of 15% due to the difference in evolutionary branching. Although the sequence conservation was limited to the EF-hand motifs in AIF-1, the overall structures of these proteins for Ca^2+^-bound states were relatively well-superimposed (RMSD of _CD_EFhd1 and _CD_EFhd2 = 0.403 Å for 85 C_α_ atoms, RMSD of _CD_EFhd1 and AIF-1 = 2.089 Å for 63 C_α_ atoms) (Figure 2A). EF-hand motifs of these proteins accommodate a helix (LM-helix in _CD_EFhd1 and _CD_EFhd2, and helix E in AIF-1), comparable with the binding mode of the calmodulin-ligand interaction (Durvanger and Harmat, 2019). When we compared the Ca^2+^-bound _CD_EFhd1 and _CD_EFhd2, the LM-helices of both proteins participated in the intramolecular hydrophobic interactions with the hydrophobic groove of the EF-hands, and the hydrophobic interaction networks of _CD_EFhd1 and _CD_EFhd2 were structurally conserved (Figures 2B,C) (Park et al., 2016). Unlike the LM-helix of _CD_EFhd1 and _CD_EFhd2, the helix E of Ca^2+^-bound AIF-1 formed intermolecular hydrophobic interactions with the hydrophobic groove of two EF-hands in a symmetry-mate molecule, resulting in the dimer formation of AIF-1 (Yamada et al., 2006). When we compared the intramolecular interactions in _CD_EFhd1 and the intermolecular interactions in AIF-1, a distinct hydrophobic interaction network between the hydrophobic groove of the EF-hands and the accommodated helix (LM-helix in _CD_EFhd1 and helix E′ in AIF-1) was found (Figures 2B,D). Collectively, the overall structure of _CD_EFhd1 was similar to that of _CD_EFhd2 and AIF-1, but the hydrophobic interaction network between the LM-helix and hydrophobic groove of EF-hands in _CD_EFhd1 was similar to that of _CD_EFhd2, but not AIF-1.

To compare the EF-hand motifs of _CD_EFhd1, _CD_EFhd2, and AIF-1, we superimposed the structures based on EF-hand 1 or 2. In _CD_EFhd1 and _CD_EFhd2, EF-hand 1 coordinated Ca^2+^, and the Ca^2+^ coordinating residues were well-superimposed (RMSD = 0.269 Å for 36 C_α_ atoms) (Figure 2E). However, the EF-hand 1 of AIF-1 could not coordinate Ca^2+^ because there was no space for Ca^2+^ coordination due to the β-turn, which was stabilized by a hydrogen bond network comprised of N60, N62, and D64 (Yamada et al., 2006) (Figure 2F). In addition, the consensus residues for the EF-hand 1 of AIF-1 were not conserved with those of _CD_EFhd1 and _CD_EFhd2 (Supplementary Figure 1). Although EF-hand 2 of these proteins could coordinate Ca^2+^, the geometries for Ca^2+^ coordination were distinct. The EF-hand 2 of EFhd1 and EFhd2 formed geometries of the pentagonal bipyramid for Ca^2+^ coordination, but that of AIF-1 formed a trigonal bipyramidal geometry (Figures 2E,F). This originated from the differences in sequences between AIF-1 and EFhd1 or EFhd2.

The canonical EF-hand domain has two helix-loop-helix motifs comprising four helices (helix 1, helix 2, helix 3, and helix 4) and forms two hydrophobic clusters (I and II) (Denessiouk et al., 2014). Helices 1 and 4 form hydrophobic cluster I, comprising three aromatic residues, and helices 2 and 3 usually form hydrophobic cluster II, comprising a combination of aromatic, hydrophobic, and polar amino acids. Based on the conformational changes of the hydrophobic clusters I and II upon Ca^2+^ binding, EF-hand-containing proteins can be classified into five separate types (Denessiouk et al., 2014). A previous report suggested that _CD_EFhd2 belongs to type I, which maintains an open conformation, secondary structures, and cluster interactions independent of Ca^2+^ (Ferrer-Acosta et al., 2013; Park et al., 2016). Although we could not determine the type of _CD_EFhd1 due to the lack of the structure of the apo-state, we expected that _CD_EFhd1 also belonged to type I because of its structure and sequence similarity with _CD_EFhd2 (Figure 2A, Supplementary Figure 2). Indeed, the structures of the EF-hand motifs comprising hydrophobic clusters were highly conserved in _CD_EFhd1 and _CD_EFhd2 [RMSDs of EF-hand motifs, cluster I, and cluster II are 0.334 Å (for 66 C_α_ atoms), 0.266 Å (for 25 C_α_ atoms), and 0.177 Å (for 22 C_α_ atoms), respectively]. In addition, the sequences of the EF-hands in both proteins were highly conserved with a sequence identity of 85% (hydrophobic cluster I: F100, F149, and F152 in EFhd1; F101, F150, and F153 in EFhd2; hydrophobic cluster II: L113, L116, and I136 in EFhd1; L114, L117, and I137 in EFhd2). Therefore, _CD_EFhd1 was classified as type I.

### Zn^2+^ Ions in the Crystal Packing Interfaces of the EFhd1 Core Domain

**Figure 3:**
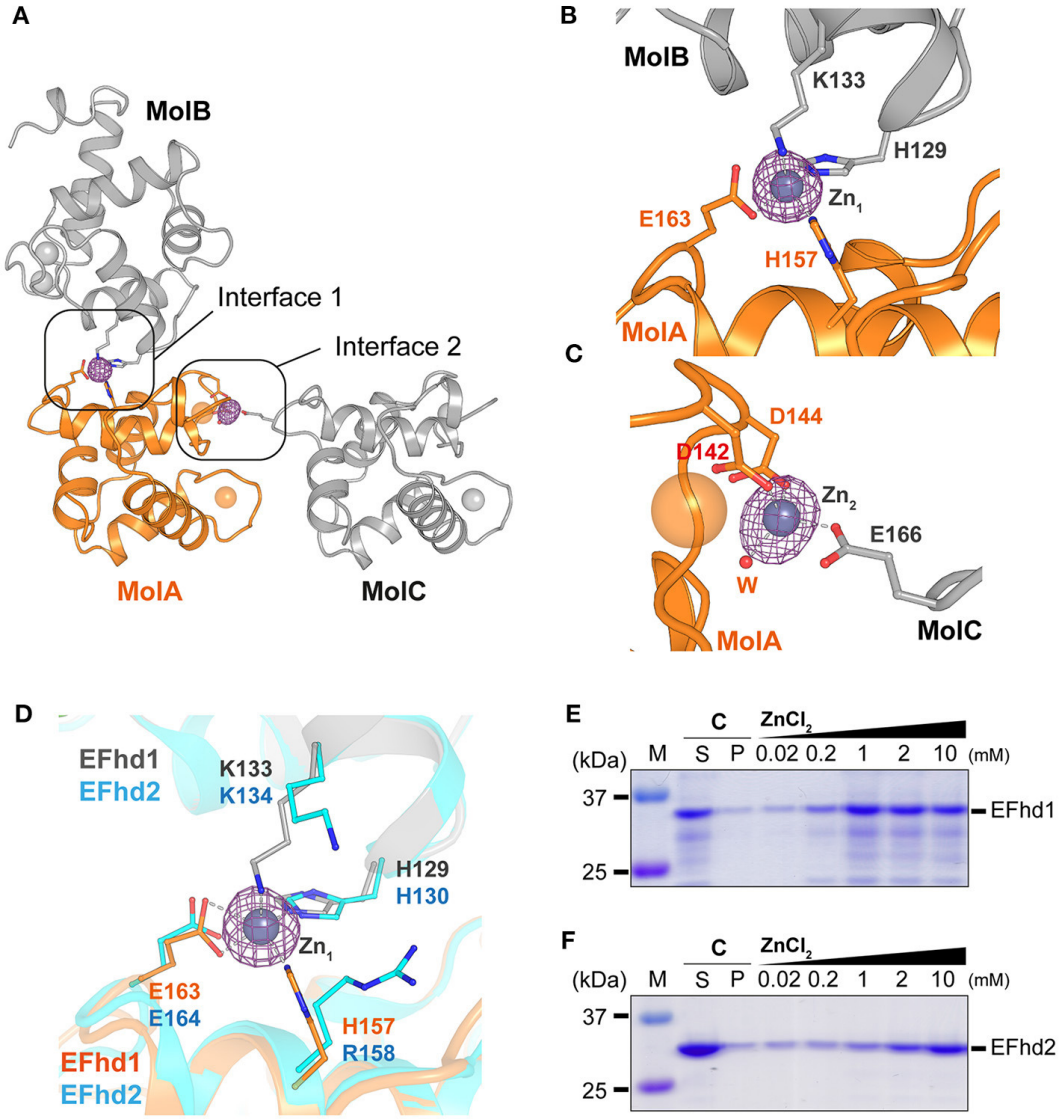
Zn^2+^-mediated crystal packing interactions of _CD_EFhd1 and Zn^2+^-dependent precipitation assay of _CD_EFhd1 and _CD_EFhd2. **(A)** A cartoon representation of the symmetry mate molecules whose interactions are mediated by Zn^2+^. Interfaces 1 and 2 are formed by MolA and MolB or MolA and MolC, respectively. MolA is colored in orange, and MolB and MolC are colored in gray. The residues for intermolecular interactions are represented in the stick form. The *F*_obs_-*F*_calc_ maps for Zn^2+^ are marked by the magenta mesh form. **(B,C)** Detailed view of interface 1 **(B)** and interface 2 **(C)**. The residues for Zn^2+^ coordination are represented in stick form. The Zn^2+^ coordination is marked by dashed lines. **(D)** Detailed view of superimposed interface 1 of EFhd1 (orange and gray) and EFhd2 (cyan) (PDB ID: 5I2L). The residues for Zn^2+^ coordination are represented in the stick form. **(E,F)** SDS-PAGE results of the Zn^2+^-dependent precipitation assay using EFhd1 **(E)** or EFhd2 **(F)**. Zn^2+^ untreated samples are marked by control (C) and were centrifuged to separate the supernatant (S) and precipitant (P) fractions. The samples mixed with various concentrations of ZnCl_2_ (0.02, 0.2, 1, 2, and 10 mM) were centrifuged to separate supernatant (S) and precipitant (P) fractions, and the P fractions were analyzed via SDS-PAGE.

In the electron density map (*F*_obs_-*F*_calc_) of _CD_EFhd1, we observed two strong unidentified electron density maps located at the interfaces of the symmetry-mate molecules (Figure 3A). We further analyzed the unidentified electron density based on the metal coordinating geometric analysis performed with the CheckMyMetal and MetalPDB server (Zheng et al., 2017; Putignano et al., 2018). The coordination geometry was predicted to be tetrahedron, which is the major geometry for Zn^2+^ coordination. In addition, with the addition of 5 mM ZnSO_4_ to the crystallization condition, we expected that Zn^2+^ ions (Zn_1_ and Zn_2_) would be present in the maps. We identified two Zn^2+^-mediated interactions between one _CD_EFhd1 (MolA) and other symmetry-mate molecules (MolB and MolC) (Figures 3B,C). Zn_1_ was coordinated by H129 (MolB), K133 (MolB), H157 (MolA), and E163 (MolA), and Zn_2_ was coordinated by D142 (MolA), D144 (MolA), a water molecule, and E166 (MolC). To further analyze the Zn^2+^-mediated interactions, we compared the interfaces between MolA and MolB (interface 1), and between MolA and MolC (interface 2). The interface areas of interfaces 1 and 2 were 339 and 151 Å^2^, respectively, and the predicted binding energy of interface 1 (−4.3 kcal mol^−1^) was lower than that of interface 2 (−3.3 kcal mol^−1^), suggesting that interface 1 is more energetically stable than interface 2. Therefore, interface 1 might contribute more to the Zn^2+^-mediated multimerization of the EFhd1 than interface 2.

The Zn_1_ coordinating residues in EFhd1 (H129, K133, H157, and E163) were highly conserved with those in EFhd2 (H130, K134, R158, and E164), except H157 in EFhd1, which was replaced by R158 in EFhd2 (Figure 3D, Supplementary Figure 1). As histidine is a major ligand for Zn^2+^, we expected that the Zn^2+^-mediated multimerization of EFhd1 would be observed to a greater degree than that of EFhd2. To evaluate the difference between the Zn^2+^-mediated multimerization of EFhd1 and EFhd2, we performed Zn^2+^-dependent precipitation assays and turbidity measurements. In the precipitation assays, we found that the precipitation ratio of EFhd1 and EFhd2 increased in proportion to [Zn^2+^] (Figures 3E,F). Consistent with the precipitation assays, the turbidities of both proteins increased with a [Zn^2+^] (Supplementary Figure 3), and we obtained the $K_{1/2}^{agg}$ (concentration of half maximal protein aggregation for Zn^2+^) of 0.41 ± 0.02 mM for EFhd1 and 5.9 ± 0.4 mM for EFhd2. Therefore, we concluded that EFhd1 and EFhd2 could be multimerized by Zn^2+^, and EFhd1 was more sensitive to Zn^2+^ than EFhd2 for multimerization.

### Actin-Binding and -Bundling Activities of EFhd1

**Figure 4:**
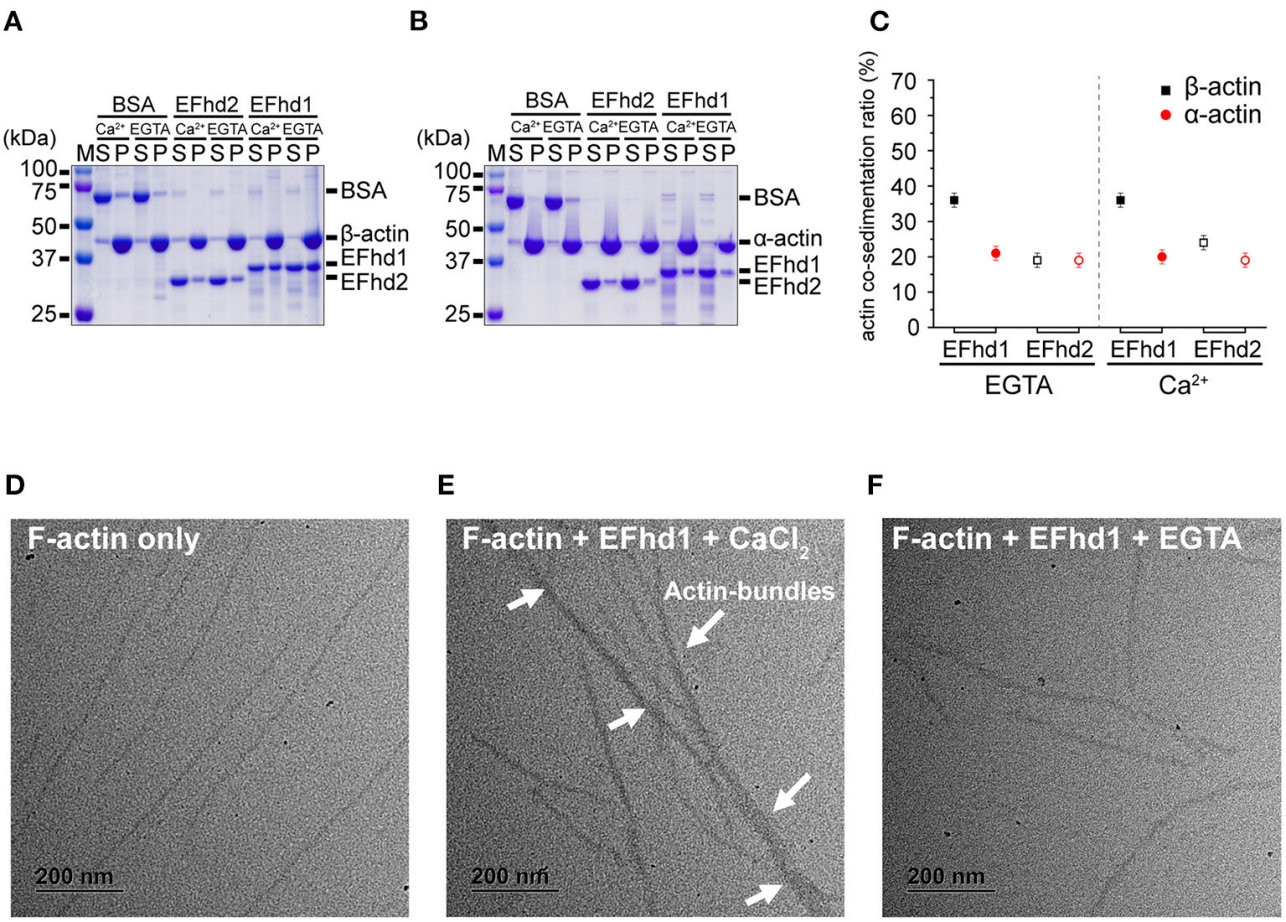
*In vitro* β/α-actin-binding and -bundling activities of EFhd1. **(A,B)** SDS-PAGE results for co-sedimentation assays with EFhd1 and filamentous β-actin **(A)** and α-actin **(B)**. EFhd2 and BSA were used as positive and negative controls, respectively. To control the presence or absence of the Ca^2+^ environment, 1 mM CaCl_2_ or 1 mM EGTA was added. **(C)** Quantification of the results of the co-sedimentation assays. The average values of the co-sedimentation ratios of each experiment are marked by black squares or red spheres. The error bars represent the 95% confidence interval for the mean, which was calculated from 10 independent experiments. Filled and open black squares represent the co-sedimentation ratios of EFhd1 and EFhd2 with β-actin, respectively. Filled and open red spheres represent the co-sedimentation ratios of EFhd1 and EFhd2 with α-actin, respectively. All co-sedimentation ratios were measured in the presence and absence of Ca^2+^. **(D–F)** Micrographs of negative staining electron microscopy for F-actin only **(D)**, F-actin with 0.5 mM CaCl_2_ **(E)**, and F-actin with EFhd1 and 1 mM EGTA **(F)**.

EFhd2 and AIF-1, which belong to the EF-hand superfamily proteins, have F-actin-binding and -bundling activities (Sasaki et al., 2001; Schulze et al., 2008; Huh et al., 2013; Kwon et al., 2013). As EFhd1 also belongs to the EF-hand superfamily proteins and has a high sequence similarity with EFhd2, we expected that EFhd1 would also be involved in F-actin-binding and -bundling. To measure the actin-binding activity, we performed *in vitro* high-speed co-sedimentation assays using β-actin with full-length EFhd1 or EFhd2 (Figure 4A). The co-sedimentation ratios of EFhd1 and EFhd2 in the presence of Ca^2+^ were 36 ± 2% (all errors of means are at 95% confidence interval) and 24 ± 2%, respectively. In the absence of Ca^2+^, the co-sedimentation ratios of EFhd1 and EFhd2 were 36 ± 2% and 19 ± 2%, respectively. Both proteins were Ca^2+^-independently co-sedimented with β-actin, but the sedimentation ratio of EFhd1 was 1.7-fold higher than that of EFhd2. This suggested that EFhd1 has a higher binding affinity for β-actin than EFhd2, independent of Ca^2+^.

While α-actin is localized in the cytosol, β-actin is localized not only in the cytosol but also in the mitochondrial matrix (Storch et al., 2007; Reyes et al., 2011; Xie et al., 2018). We hypothesized that the binding affinity of EFhd1 for β-actin may differ from that of α-actin because EFhd1 is localized in mitochondria (Tominaga et al., 2006). We performed co-sedimentation assays using α-actin with full-length EFhd1 or EFhd2 (Figure 4B). The co-sedimentation ratios of EFhd1 and EFhd2 in the presence of Ca^2+^ were 20 ± 2% and 19 ± 2%, respectively. In the absence of Ca^2+^, the co-sedimentation ratios of EFhd1 and EFhd2 were 21 ± 2% and 19 ± 2%, respectively. Both proteins were similarly co-sedimented with α-actin independent of Ca^2+^, and the sedimentation ratio of EFhd1 was comparable to that of EFhd2, suggesting similar α-actin-binding affinities of EFhd1 and EFhd2. When we compared the sedimentation ratio of EFhd1 for α- and β-actin, the sedimentation ratio for β-actin was 1.8-fold higher than that for α-actin, suggesting that EFhd1 has higher binding affinities for β-actin than α-actin. In the case of EFhd2, the sedimentation ratio for α- and β-actin was similar, suggesting similar binding affinities of EFhd2 for α- and β-actin. Collectively, EFhd1 had a higher actin-binding affinity for β-actin than that for α-actin regardless of Ca^2+^, and EFhd2 had a similar actin-binding affinity for α- and β-actin independent of Ca^2+^ (Figure 4C).

In addition to the co-sedimentation assays, we performed negative staining electron microscopy imaging with EFhd1 and F-actin (β-actin) to identify the actin-bundling activity of EFhd1 (Figures 4D–F). In the electron micrographs, we found the F-actin bundles only in the presence of Ca^2+^, suggesting the Ca^2+^-dependent actin-bundling activity of EFhd1. Meanwhile, the F-actin-bundling activity of EFhd1 for β-actin was lower than that of EFhd2 for α-actin (Huh et al., 2013). Collectively, EFhd1 had a Ca^2+^-dependent β-actin-bundling activity, which is lower than the α-actin-bundling activity of EFhd2.

## Discussion

This study demonstrated the crystal structure of the EFhd1 core domain, whose overall structure was similar to that of EFhd2 and AIF-1. We found two Zn^2+^ ions in the crystal packing interface, providing new insights into the Zn^2+^-mediated multimerization of EFhd1. In addition, we first identified the actin-binding and -bundling activities of EFhd1 *in vitro*. For β-actin, EFhd1 had Ca^2+^-independent β-actin-binding and Ca^2+^-dependent β-actin-bundling activities. EFhd1 bound not only to β-actin, but also to α-actin *in vitro*, which is the primary actin isoform in the cytosol, implying that EFhd1 could bind to α-actin in the cytosol.

We identified the Zn^2+^-mediated aggregation of EFhd1 with $K_{1/2}^{agg}$ = 0.41 ± 0.02 mM. Mitochondrial [Zn^2+^] remains controversial, but it is estimated to be in the submicromolar range in the Zn^2+^ overload state (Sensi et al., 2003; Park et al., 2012; Chabosseau et al., 2014). Although [Zn^2+^] for half aggregation of EFhd1 *in vitro* was much higher than that of mitochondrial [Zn^2+^], and the concentration of EFhd1 may differ between *in vitro* and physiological conditions, we cannot rule out the possibility of the multimerization of EFhd1 in the Zn^2+^ overload state because the local spatial and temporal mitochondrial [Zn^2+^] may be much higher than the reported micromolar range. It will be interesting to study the structural and functional role of Zn^2+^ ions in actin-binding and -bundling activities. In the cytosol, [Zn^2+^] is tightly regulated from the picomolar to nanomolar range, suggesting that the Zn^2+^-mediated multimerization of EFhd1 may not occur in the cytosol (Kambe et al., 2015). Thus, we suggest that EFhd1 binds to actin and is multimerized by Zn^2+^ in the mitochondria.

EFhd1 KO neurons showed alterations in mitochondrial morphology to a shortened shape, and the mitochondrial morphology could be affected by β-actin regulation (Xie et al., 2018; Ulisse et al., 2020). We found that EFhd1 had Ca^2+^-independent β-actin-binding and Ca^2+^-dependent β-actin-bundling activities (Figure 4). Therefore, we suggest that EFhd1 binds to β-actin in the resting state and induces β-actin-bundling in the Ca^2+^ overload state of mitochondria. The regulation of β-actin not only affects mitochondrial morphology but also the energy synthesis of mitochondria. The energy synthesis of mitochondria is reduced when the expression of *efhd1* is downregulated (Stein et al., 2017; Ulisse et al., 2020). Therefore, we suggest that EFhd1 induces actin rearrangement in the mitochondria, resulting in changes in energy synthesis.

In this study, we determined the crystal structure of mouse EFhd1 without C-terminal coiled-coil, and proposed Zn^2+^-mediated EFhd1 multimerization. In addition, we unveiled the actin-binding and -bundling activities of EFhd1. Nevertheless, the C-terminal coiled-coil is important for understanding the actin regulation mechanism of EFhd1. The coiled-coil of EFhd1 is expected to be important for the dimerization of EFhd1 and actin-bundling activity because these regions are highly conserved in EFhd1 and EFhd2 (Kwon et al., 2013). Therefore, to understand the structural and functional role of EFhd1, structural studies on the full-length EFhd1 and EFhd1-actin filament complexes need to be performed.

## Accession Number

Atomic coordinates and structure factors of _CD_EFhd1 have been deposited in the RCSB PDB with accession code 7CLT.

## Data Availability Statement

The datasets presented in this study can be found in online repositories. The names of the repository/repositories and accession number(s) can be found at: http://www.wwpdb.org/, 7CLT.

## Author Contributions

SHE, C-DJ, and SAM planned and organized the experiments. SAM and JYK carried out the gene cloning and expression. SAM performed the purification, crystallization, structure determination and analysis, ensemble refinement, data analysis, *in vitro* actin co-sedimentation assay, and analysis of electron microscopy. KRP performed *in vitro* actin co-sedimentation assay and electron microscopy analysis. YL and JP collected the X-ray diffraction data. TP, MJ, and JY performed the structure determination. SHE, SAM, and JP wrote the manuscript with critical editorial input from the C-DJ. All authors contributed to the article and approved the submitted version.

## Conflict of Interest

The authors declare that the research was conducted in the absence of any commercial or financial relationships that could be construed as a potential conflict of interest.
