# Supplementary material for: Structural and Biochemical Characterization of EFhd1/Swiprosin-2, an Actin-Binding Protein in Mitochondria
Source: Front Cell Dev Biol. 2021 Jan 18;8:628222. doi: 10.3389/fcell.2020.628222 (PMC7848108; doi:10.3389/fcell.2020.628222)
Supplement: Supplementary file 1 [file Table_1.DOCX]

Supplementary Material


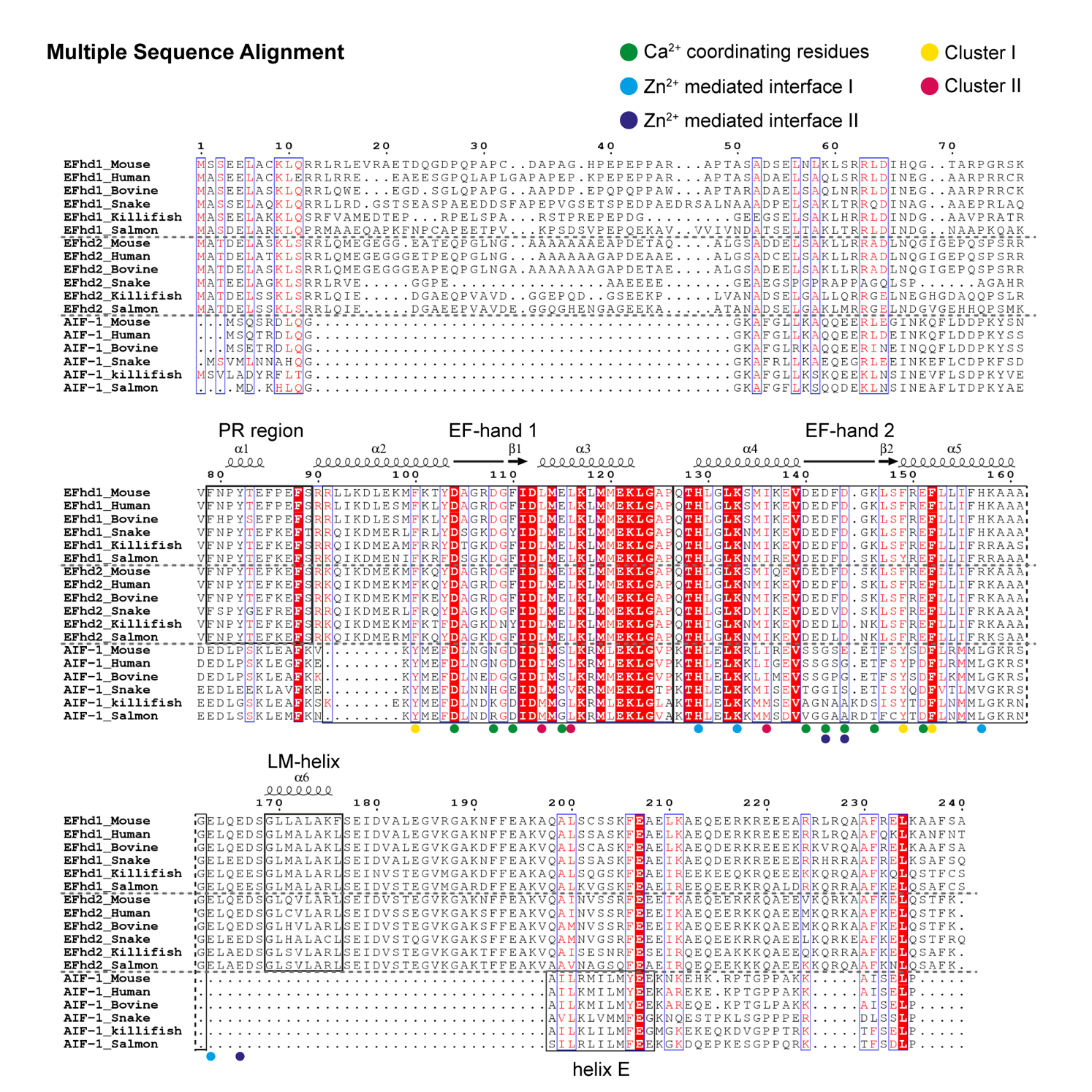


**Supplementary Figure 1. Multiple sequence alignment of EFhd1, EFhd2, and AIF-1.**

Amino acid sequence alignment of EFhd1, EFhd2, and AIF-1 sequences from various species. Key residues for Ca^2+^ coordination are marked by green dots. The residues comprising the Zn^2+^-mediated interfaces I and II are marked by cyan and blue dots, respectively. The hydrophobic clusters I and II in the EF-hand motifs are marked by yellow and magenta dots, respectively.

**
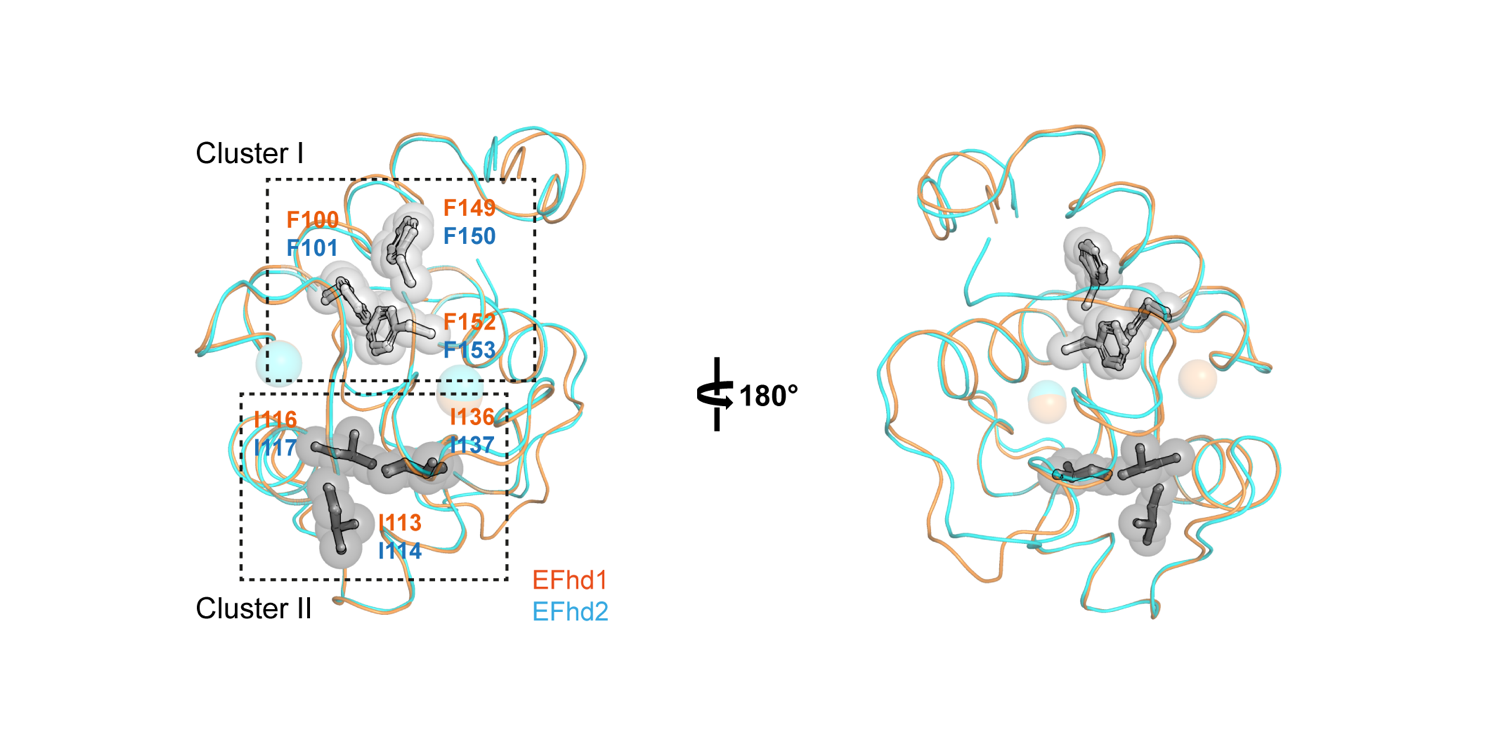
**

**Supplementary Figure 2. Hydrophobic clusters of EFhd1 and EFhd2.**

Detailed view of the superimposed hydrophobic clusters in EFhd1 and EFhd2. The residues forming the hydrophobic clusters are presented in the stick and sphere form. The hydrophobic cluster I in EFhd1 (F100, F149, F152) or EFhd2 (F101, F150, F153) is colored in gray, and the hydrophobic cluster II in EFhd1 (I113, I116, I136) or EFhd2 (I114, I117, I137) is colored in dark gray.


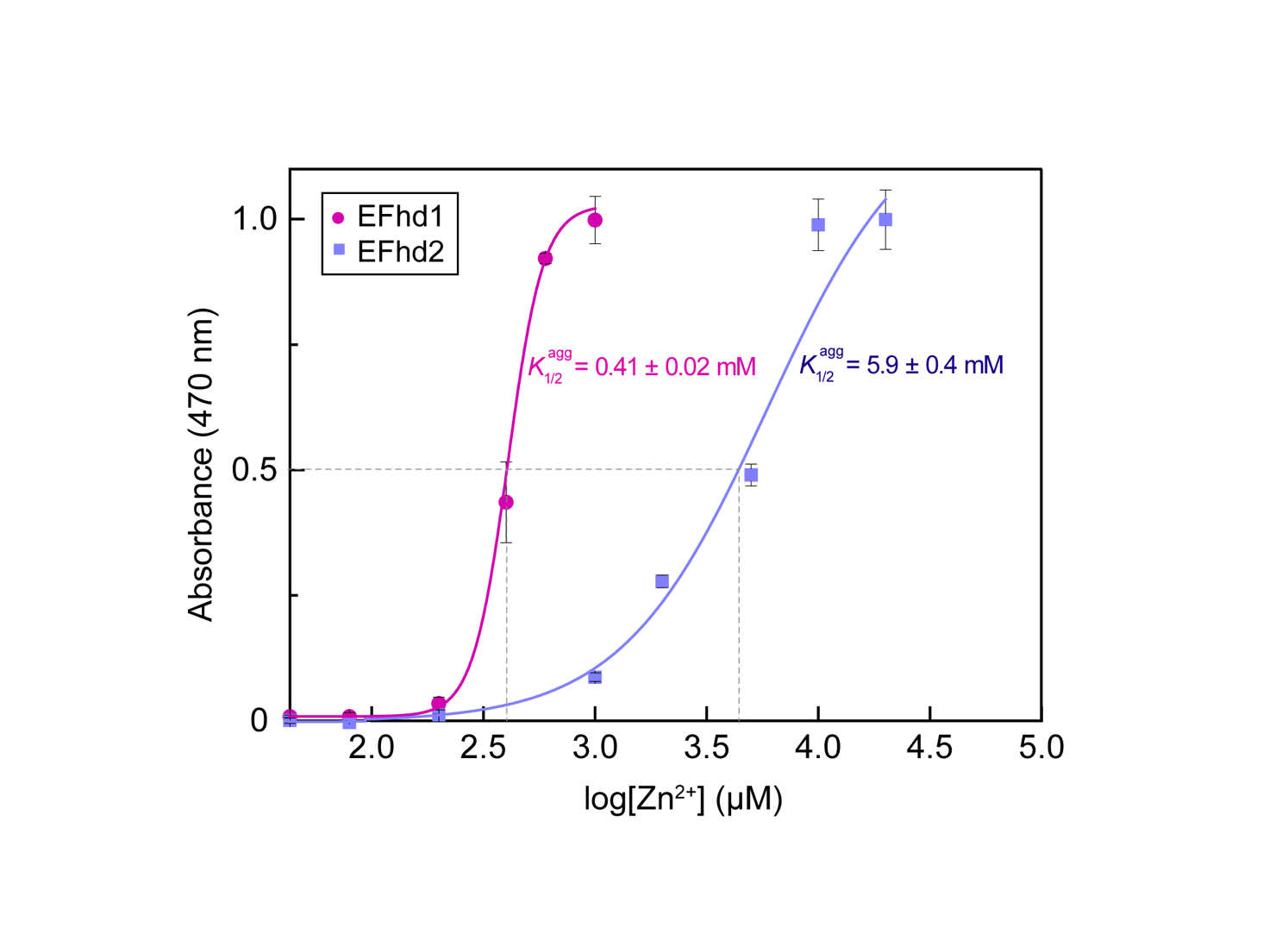


**Supplementary Figure 3. Zn^2+^-dependent precipitation assay.**

Absorbance at a wavelength of 470 nm for EFhd1 (magenta) or EFhd2 (blue) according to [Zn^2+^]. The error bars represent the 95% confidence interval for the mean calculated from three independent experiments.
